# Multiplex immunoassay analysis of biomarkers in clinically accessible quantities of human aqueous humor

**Published:** 2009-01-14

**Authors:** Rajesh K. Sharma, Anna T. Rogojina, K.V. Chalam

**Affiliations:** 1 Department of Ophthalmology, University of Florida, Jacksonville, FL; 2 St. Jude Children’s Research Hospital, Memphis, TN

## Abstract

**Purpose:**

Aqueous humor is intimately related to the cells of the anterior and posterior chambers, which affect its composition. Aqueous analysis provides useful information regarding physiological and pathophysiological processes in the eye. Human aqueous samples are typically less than 100 µl, limiting the usefulness of the analysis with traditional Enzyme-Linked immunoSorbant Assay (ELISA) techniques. The specific aim of this study was to investigate if whether large numbers of analytes can be identified in clinically available samples of aqueous humor and to document the detectability of certain biomarkers in the aqueous.

**Methods:**

We used a technology developed by Luminex xMAP to analyze hundreds of analytes in a small sample. Aqueous from eight normal and two diabetic patients was analyzed.

**Results:**

Of the 90 analytes evaluated, 52 (57%) were detectable in the normal aqueous. To place these results in biological context, we analyzed the list of expressed analytes using the MetaCore database. The functional pathways, networks, biological processes, and disease processes that these analytes represented were identified. Several ocular pathology-related processes were represented in the aqueous. The detected analytes represented biomarkers of several relevant disease processes including vascular diseases, arteriosclerosis, ischemia, necrosis, and inflammation. To provide the proof of principle that the aqueous profile could offer useful information about the pathophysiological processes, we analyzed two aqueous samples from diabetic patients. These limited samples showed the differences between normal and diabetic samples, including those relevant to diabetic retinopathy such as vascular endothelial growth factor (VEGF), C reactive protein, glutathione, and cytokines. Several biomarker groups for disease processes relevant to diabetes were perturbed.

**Conclusions:**

These results demonstrate that multiplex analysis of the aqueous can be a useful tool in screening for any pathophysiological changes of the ocular environment. Moreover, ocular pathology/pathophysiology-specific Multi-analyte profiles MAPs can be developed and used to analyze the aqueous.

## Introduction

Aqueous humor, a product of the ciliary process, occupies the anterior and posterior chambers of the eye. It supplies nutrients to the nonvascularized cornea, lens, and trabecular meshwork. It is drained through two major pathways, the iridocorneal and the uveoscleral outflows I mean the constitution of aqueous humor affects the functioning of cells, and also the functioning of cells affects the aqueous composition. In addition, functional barriers between the anterior and posterior segments are not strict. The composition of the aqueous is also likely to be affected by the physiology/pathophysiology of the retina. Consequently, several growth factors have been detected in the aqueous humor, and the composition of these proteins changes dramatically in different ocular conditions, especially in inflammation and glaucoma [1,2].

Evaluation of the aqueous composition can be a powerful tool in understanding the pathophysiology and treatment response to many ocular conditions. Assessment of cytokines in uveitis patients revealed the presence of Interleukin (IL)-2, 6, 10, 12, Interferon (IFN)-γ, tumor growth factor (TGF)-β2, tumor necrosis factor (TNF)-α, and macrophage migration inhibitory factor. One major limitation of testing the aqueous is that only small sample volumes can be obtained from human eyes. Typically 50-150 µl of aqueous can be obtained from one human eyes, which is barely sufficient to test a few cytokines using traditional ELISA techniques including sequential ELISA. Alternatively, the samples can be pooled, but this can mask individual patient differences. However, with the introduction of flow cytometric bead-based technology, multiple cytokine analytes can now be quantified simultaneously and rapidly in individual samples. This technique has better reproducibility and sensitivity than the traditional ELISA [3]. The cytometric bead array (CBA) system has proven effective in testing samples from cell culture supernatant, human serum, tears, and nasal lavage [4]. In this pilot study, we investigated the detectability of an array of proteins in the aqueous humor obtained from normal eyes to determine if this analysis could be useful in understanding disease processes and treatment responses within the eye.

The aim of this study was to investigate whether large numbers of analytes can be identified in clinically available aqueous samples. Large numbers of analytes are needed to perform global analysis for identifying maps, networks, pathways, and disease processes. Such an analysis would be useful to compare ocular environment in different conditions. Another aim of this study was to document the detectability of certain biomarkers in the aqueous.

## Methods

### Samples

Aqueous samples were collected from eight non-diabetic and 2 diabetic patients undergoing cataract surgery. Aqueous humor (80–100 µl) was withdrawn through a limbal paracentesis site using a 27 gauge needle in a tuberculin syringe. Care was taken to avoid touching intraocular tissues and to prevent contamination of aqueous samples with blood. The samples were immediately frozen and stored at −80 °C. Patients with other ocular or systemic diseases such as inflammatory diseases were excluded from the study. Informed consent was obtained from the patients, and the research was in compliance with the tenets of the University of Florida and the Declaration of Helsinki for experiments involving human tissue.

### Multiplex analysis

Multiplex analysis was performed at Rules-Based Medicine (Austin, TX), which uses Multi-Analyte Profiles (MAPs) based on powerful Luminex xMAP® technology to discover biomarker patterns within very small sample volumes. The aqueous samples were thawed at room temperature, vortexed, and then spun at 13,000x g for 5 min to remove any precipitates. Maximum available volume (80–100 µl) was removed and placed into a master microtiter plate for MAP antigen analysis. Using automated pipetting, an aliquot of each sample was introduced into one of the capture microsphere multiplexes of the human antigen MAP. The sample and capture microspheres were thoroughly mixed and incubated at room temperature for 1 h. Multiplexed cocktails of biotinylated reporter antibodies for each multiplex were then added robotically and thoroughly mixed. After this, the mixture was incubated for an additional hour at room temperature. Multiplexes were developed using an excess of streptavidin-phycoerythrin solution, which was thoroughly mixed into each multiplex and incubated for 1 h at room temperature. The volume of each multiplexed reaction was reduced by vacuum filtration, and the volume increased by dilution into a matrix buffer for analysis. Analysis was performed in a Luminex 100 instrument (Luminex, Austin, TX), and the resulting data stream was interpreted using proprietary data analysis software (developed at Rules-Based Medicine and licensed to Qiagen Instruments, Qiagen Inc., Valencia, CA). For each multiplex, calibrators and controls were both included on each microtiter plate. Eight-point calibrators were run in the first and last column of each plate, and three-level controls were included in duplicate. Testing results were first determined for the high, medium, and low controls of each multiplex to ensure proper assay performance. Unknown values for each of the analytes that localized in a specific multiplex were determined using weighted and non-weighted curve fitting algorithms included in the data analysis package.

### Analysis

The value for each analyte was obtained as a concentration (example; mg/ml). The values of eight samples (or less if an analyte could not be analyzed in all samples because of insufficient quantity of the sample) were averaged and compared to the sensitivity of the system. An analyte was considered “detectable” if the levels exceeded the minimal detectable levels.

The detectable analytes were further analyzed to identify biological/disease processes and the involved pathways/networks they participated in by using MetaCore™ analysis software (GeneGo Inc., St. Joseph, MI). The manually annotated database includes over 160,000 human protein interactions and metabolic reactions. The whole data set of 52 detectable analytes was imported in MetaCore to build an analysis of functional ontologies including GeneGo process, GeneGo disease process, canonical pathway maps, and networks. Calculation of statistical significance throughout MetaCore for maps, networks, and processes are based on p value, which are calculated based on hypergeometric distribution. P values essentially represent the probability of particular mapping arising by chance given the numbers of genes in the set of all genes on maps/networks/processes, genes on a particular map/network/process, and genes in the experiment [5]. We used a p value of 0.05 for the cutoff. The degree of relevance to different categories for the uploaded data sets is defined by p values, so that the lower p value obtains higher priority.

The experimental data were input to build networks. The three different scoring functions used to rank the small subnetworks created by the network building algorithms were zScore, gScore, and p value. The zScore ranks the subnetworks (within the analyzed network) with regards to their saturation with genes from the experiment. A high zScore means the network is highly saturated with genes from the experiment. In other words, it means that relatively larger number of genes/analytes in a particular network were present in the aqueous sample. Each network is comprised of canonical pathways. The gScore modifies the zScore based on the number of canonical pathways used to build the network. If a network has a high gScore, it is saturated with expressed genes (from the zScore), and it contains many canonical pathways.

## Results

### General analysis

A total of 90 predetermined analytes were analyzed. All 90 analytes could be analyzed in at least one sample from normal patients. In normal samples, 57% (52 out of 90) of the analytes were detectable (above the sensitivity of the method), and 42% of analytes (38 out of 90) were undetectable either because their values were below the sensitivity of the method (28 out of 90; 31%) or their quantities were insufficient to assign a value (10 out of 90; 11%). A list of detectable proteins is provided in Table 1.

**Table 1:** List of analytes detected in aqueous.

**Number**	**Analyte**	**SwissProt ID**	**Unit**	**Average**	**Standard deviation**	**Folds above detectable limits**
1	Alpha-1 Antitrypsin	P01009	mg/ml	0.00274	0.000893	52033.62
2	Adiponectin	Q15848	μg/ml	0.01054	0.002623	10.545
3	Alpha-2 Macroglobulin	P01023	mg/ml	0.0006	0.000777	2.07352
4	Alpha-Fetoprotein	P02771	ng/ml	0.33637	0.072147	3.911337
5	Apolipoprotein A1	P02647	μg/ml	0.72	0.918	22077.51
6	Apolipoprotein CIII	P02656	μg/ml	0.023	0.009896	1733.333
7	Apolipoprotein H	P02749	μg/ml	0.61937	0.554624	14076.7
8	Beta-2 Microglobulin	P61769	μg/ml	0.18121	0.126304	2745.644
9	Complement 3	P01024	mg/ml	0.00161	0.00162	61454.37
10	Cancer Antigen 125	Q14596	U/ml	9.83	11.01929	11.64692
11	Cancer Antigen 19–9	Q9BXJ9	U/ml	0.11567	0.048019	2.351045
12	CD40	P27512	ng/ml	0.03338	0.016927	7.949405
13	C Reactive Protein	P02741	μg/ml	0.01163	0.011371	1520.686
14	Endothelin-1	P05305	pg/ml	3.867	1.675357	2.693245
15	EN-RAGE	P80511	ng/ml	0.050		10.06
16	Eotaxin	P51671	pg/ml	11.737	3.148795	1.431402
17	Erythropoietin	P01588	pg/ml	47.02	54.47922	1.416416
18	Ferritin	P02792	ng/ml	3.10333	0.5231	443.3333
19	FGF basic	P09038	pg/ml	20.66	5.233832	1.054082
20	Fibrinogen	P02671	mg/ml	0.00021	0.000133	4374.364
21	G-CSF	P09919	pg/ml	1.26		1.26
22	Glutathione S-Transferase	P09488	ng/ml	0.86112	0.971832	10.65749
23	Haptoglobin	P00739	μg/ml	0.5	0.476	415.6686
24	IgA	P24071	μg/ml	0.97	0.85	23278.44
25	IGF-1	P01343	ng/ml	3.7295	4.838829	4.661875
26	IgM	P20769	mg/ml	4.82e−5	2.21e−5	638.4106
27	IL-1ra	P18510	pg/ml	5.81		1.936667
28	IL-6	P05231	pg/ml	5.81	7.328891	2.381148
29	IL-8	P10145	pg/ml	4.9412	2.029345	7.038818
30	Leptin	P41159	ng/ml	0.11360	0.178015	5.514887
31	Lipoprotein (a)	P08519	μg/ml	0.0637	0.043628	3.445946
32	MCP-1	P13500	pg/ml	308.87	191.7174	29.69952
33	MIP-1alpha	P10147	pg/ml	7.1362	2.41117	2.744712
34	MIP-1beta	P13236	pg/ml	21.707	13.95261	2.863786
35	MMP-3	P08254	ng/ml	0.22912	0.227617	5.728125
36	Myoglobin	P02144	ng/ml	0.924	0.331162	176
37	PAI-1	P05121	ng/ml	0.62833	0.483399	139.6296
38	Prostatic Acid Phosphatase	P15309	ng/ml	0.01419	0.00844	2.075815
39	PAPP-A	Q13219	U/ml	0.01645	0.011151	2.223057
40	“Prostate Specific Antigen, Free”	P07288	ng/ml	0.0051		1.094421
41	RANTES	P13501	ng/ml	0.00233	0.001534	9.668737
42	Serum Amyloid P	P02743	μg/ml	0.0096	0.003076	841.7391
43	Stem Cell Factor	P21583	pg/ml	20.575	4.99135	1.85027
44	SGOT	P17174	μg/ml	1.0505	0.466578	1.411962
45	SHBG	P04278	nmol/l	0.1696	0.070577	652.3077
46	Thyroxine Binding Globulin	P05543	μg/ml	0.29598	0.26957	4339.993
47	Tissue Factor	P13726	ng/ml	0.4732	0.156952	2.813615
48	TIMP-1	P01033	ng/ml	13.742	11.56826	327.5924
49	TNF RII	Q92956	ng/ml	0.07006	0.051629	107.7949
50	Thyroid Stimulating Hormone	P01215	U/ml	0.0217	0.018148	3.882143
51	VCAM-1	P19320	ng/ml	2.22	1.408971	170.7692
52	VEGF	P15692	pg/ml	214.987	91.01978	143.325

Out of 90 analytes evaluated, 52 were detectable in the aqueous samples. These included cytokines, growth factors, cell adhesion molecules immunoglobulin, components of immune system, and enzymes. VEGF is especially relevant for studying the pathobiology of various retinal disorders.

### Functional analysis

The detected analytes were evaluated by MetaCore software to identify possible relations among proteins. The accession numbers (SwissProt IDs) for the analytes in Table 1 were identified and uploaded into the MetaCore software. This software generates networks based on interactions between uploaded proteins and all others proteins/genes stored in the knowledge database. For each network, the probability of finding the uploaded proteins in the network by chance was calculated. The negative logarithm of the probability represents how relevant this network was to the list of uploaded proteins.

#### Distribution by canonical pathways maps

Canonical pathway maps represent a set of approximately 500 signaling and metabolic maps covering human biology in a comprehensive way. Of the 52 proteins identified in the normal aqueous, 35 were present in the MetaCore database on maps. The 10 most significant canonical pathway maps that are represented relate to immune response, development, and cell adhesion (Table 2).

**Table 2:** List of 10 most significant canonical pathways maps represented by detectable analytes in normal aqueous samples.

**Number**	**Name**	**p value**
1	Immune response _Histamine signaling in dendritic cells	1.098e−7
2	Development_Leptin signaling via JAK/STAT and MAPK cascades	1.194e−7
3	Immune response _Oncostatin M signaling via JAK-Stat in mouse cells	1.335e−6
4	Immune response _Oncostatin M signaling via JAK-Stat in human cells	2.099e−6
5	Immune response _Histamine H1 receptor signaling in immune response	2.881e−6
6	Cell adhesion_ECM remodeling	3.911e−6
7	Immune response _MIF-mediated glucocorticoid regulation	1.501e−4
8	Immune response _IL1 signaling pathway	5.128e−4
9	Immune response _Oncostatin M signaling via MAPK in mouse cells	6.111e−4
10	Immune response _Oncostatin M signaling via MAPK in human cells	7.207e−4

These pathways prominently represent immune and cell adhesion pathways.

#### Most relevant networks

The list of detectable analytes was used to generate biological networks using the Analyze Networks (AN) algorithm, which is a variant of the shortest paths algorithm, with main parameters of (1) relative enrichment with the uploaded data and (2) relative saturation of networks with canonical pathways. Subnetworks are ranked by a p value and gScore and interpreted in terms of Gene Ontology. All 52 proteins identified were present in the MetaCore database on networks (Table 3).

**Table 3:** List of 10 most statistically significant canonical pathways maps represented by detectable analytes in normal aqueous samples.

**Number**	**Key network objects**	**GO Processes**	**p value**	**zScore**	**gScore**
1	A2M, Kallikrein 3 (PSA), Alpha 1-antitrypsin, VEGF-A, PAI1	organ development (61.4%; 4.075e−10), response to wounding (36.4%; 4.455e−10), response to external stimulus (43.2%; 4.572e−10), cell proliferation (50.0%; 5.309e−10), positive regulation of cell proliferation (31.8%; 6.986e−10)	2.36e−31	61.1	61.1
2	Stromelysin-1, CCL2, TIMP1, MIP-1-beta, Alpha 1-antitrypsin	response to external stimulus (47.8%; 1.468e−12), response to wounding (37.0%; 9.322e−11), wound healing (23.9%; 1.290e−10), blood coagulation (15.2%; 3.146e−07), coagulation (15.2%; 3.914e−07)	8.46e−29	57.4	57.4
3	VEGF-A, Endothelin-1, PAI1, Stromelysin-1, CD40(TNFRSF5)	response to stimulus (75.5%; 5.440e−14), response to stress (55.1%; 3.472e−13), protein kinase cascade (40.8%; 3.739e−13), response to external stimulus (46.9%; 7.466e−13), cell surface receptor linked signal transduction (57.1%; 1.494e−12)	1.14e−28	56.8	56.8
4	F3, Beta-2-glycoprotein I (APOH), VCAM1, MIP-1-beta, CCL2	cell adhesion (53.2%; 8.293e−19), biological adhesion (53.2%; 8.293e−19), response to wounding (51.1%; 1.218e−18), localization of cell (51.1%; 5.939e−17), cell motility (51.1%; 5.939e−17)	1.14e−28	56.8	58.1
5	CD40(TNFRSF5), CRP, G-CSF, F3, APCS	immune system process (57.1%; 2.349e−16), immune response (44.9%; 1.384e−14), response to stimulus (73.5%; 4.961e−13), defense response (40.8%; 6.493e−12), adaptive immune response (20.4%; 1.681e−10)	1.14e−28	56.8	56.8
6	APOA1, PPAP, Adiponectin, APOC3, MIP-1-alpha	sterol transport (15.2%; 3.842e−08), cholesterol transport (15.2%; 3.842e−08), triacylglycerol metabolic process (12.1%; 1.119e−06), lipid transport (15.2%; 1.661e−06), response to stimulus (63.6%; 1.781e−06)	4.46e−27	57.2	57.2
7	CCL2, CCL5, Eotaxin, MIP-1-beta, MIP-1-alpha	cytokine and chemokine mediated signaling pathway (25.5%; 4.692e−17), locomotory behavior (38.3%; 1.594e−16), chemotaxis (31.9%; 5.793e−16), taxis (31.9%; 5.793e−16), response to external stimulus (53.2%; 1.704e−15)	1.82e−23	48	68
8	A2M, Endothelin-1, CCL5, IL-6, MIP-1-beta	response to stimulus (75.5%; 5.440e−14), response to external stimulus (49.0%; 7.073e−14), regulation of protein metabolic process (34.7%; 5.809e−13), defense response (42.9%; 6.006e−13), inflammatory response (34.7%; 1.171e−12)	5.95e−21	43.6	43.6
9	Beta-2-microglobulin, APOA1, TR2(TNFRSF14), VCAM1, GSTM1	response to stimulus (75.0%; 1.873e−11), positive regulation of transferase activity (27.5%; 2.304e−10), positive regulation of kinase activity (25.0%; 3.531e−09), positive regulation of catalytic activity (27.5%; 2.827e−08), regulation of transferase activity (27.5%; 3.249e−08)	4.09e−16	34.9	34.9
10	PPAP, Thyroxine-binding globulin, Adiponectin, Epo, APOLPA	protein kinase cascade (34.3%; 2.065e−07), intracellular signaling cascade (48.6%; 2.204e−07), positive regulation of kinase activity (22.9%; 2.930e−07), positive regulation of transferase activity (22.9%; 3.458e−07), regulation of cell differentiation (25.7%; 3.570e−07)	2.29e−14	33.3	33.3

Network objects represent the fraction of the number of genes from input list against total number of genes from MetaCore database for respective network. These included cell adhesion pathways, cytokine signaling pathways, and pathways for immune system processes.

#### Distribution by GeneGo and Gene Ontology processes

The content of about 110 cellular and molecular processes is defined and annotated by GeneGo. Each process represents a preset network of protein interactions characteristic to the process. Of the 52 proteins identified, 41 were present in the MetaCore database on GeneGo processes. The top 10 processes represented in the normal aqueous samples are given in Table 4. These processes mostly represented responses to stimuli, immune processes, response to wounding, inflammation, defense and stress, cell proliferation, and chemotaxis.

**Table 4:** List of 10 most statistically significant GeneGO processes represented by detectable analytes in normal aqueous samples.

**Number**	**Process**	**p value**
1	Response to external stimulus	1.63e−11
2	Response to stimulus	2.9e−11
3	Immune system process	2.41e−9
4	Response to wounding	1.02e−8
5	Inflammatory response	1.04e−7
6	Defense response	1.36e−7
7	Immune response	3.86e−7
8	Response to stress	6.83e−7
9	Positive regulation of cell proliferation	7.37e−6
10	Chemotaxis	1.47e−5

Some of these processes, such as response to stress, chemotaxis, regulation of cell proliferation, wound healing, and immune system processes are relevant in ocular diseases.

#### Distribution by disease biomarkers

There are over 500 human diseases with gene content annotated by GeneGo and organized in disease folders, which are further organized into a hierarchical tree. Certain factors may affect p value prioritization for diseases. For example, the gene content may vary greatly between complex diseases (such as cancers and some Mendelian diseases), and also, the coverage of different diseases in the literature may be skewed. Of the 52 proteins identified, 50 were present in the MetaCore database on diseases. The diseases whose biomarkers were detectable in the aqueous are relevant conditions of the retina including vascular (together with vascular occlusive and cardiovascular diseases), arteriosclerosis, ischemia, necrosis, and inflammation. A list of top 10 disease processes represented is given in Table 5.

**Table 5:** List of 10 most statistically significant disease biomarkers represented by detectable analytes in normal aqueous samples.

**Number**	**Name**	**p value**
1	Vascular Diseases	1.843e−21
2	Arterial Occlusive Diseases	5.575e−21
3	Cardiovascular Diseases	4.352e−20
4	Arteriosclerosis	5.866e−20
5	Necrosis	1.571e−18
6	Ischemia	9.303e−18
7	Myocardial Ischemia	1.746e−16
8	Coronary Disease	1.269e−15
9	Infection	1.637e−15
10	Lung Diseases, Obstructive	2.774e−15

Biomarkers for vascular diseases, ischemia, necrosis,  and infection are highly relevant for ocular pathology.

### Comparison between non-diabetic and diabetic aqueous samples

To provide the proof of principle that functional analysis of the aqueous using multiplex technology could provide useful information and identify differences between normal and disease conditions, we also performed the analysis on two diabetic samples. Out of 90 analytes, 44 (49%) were detected and 33 were not detected (36%). Quantity was not sufficient to analyze 13 analytes.

**Figure 1:**
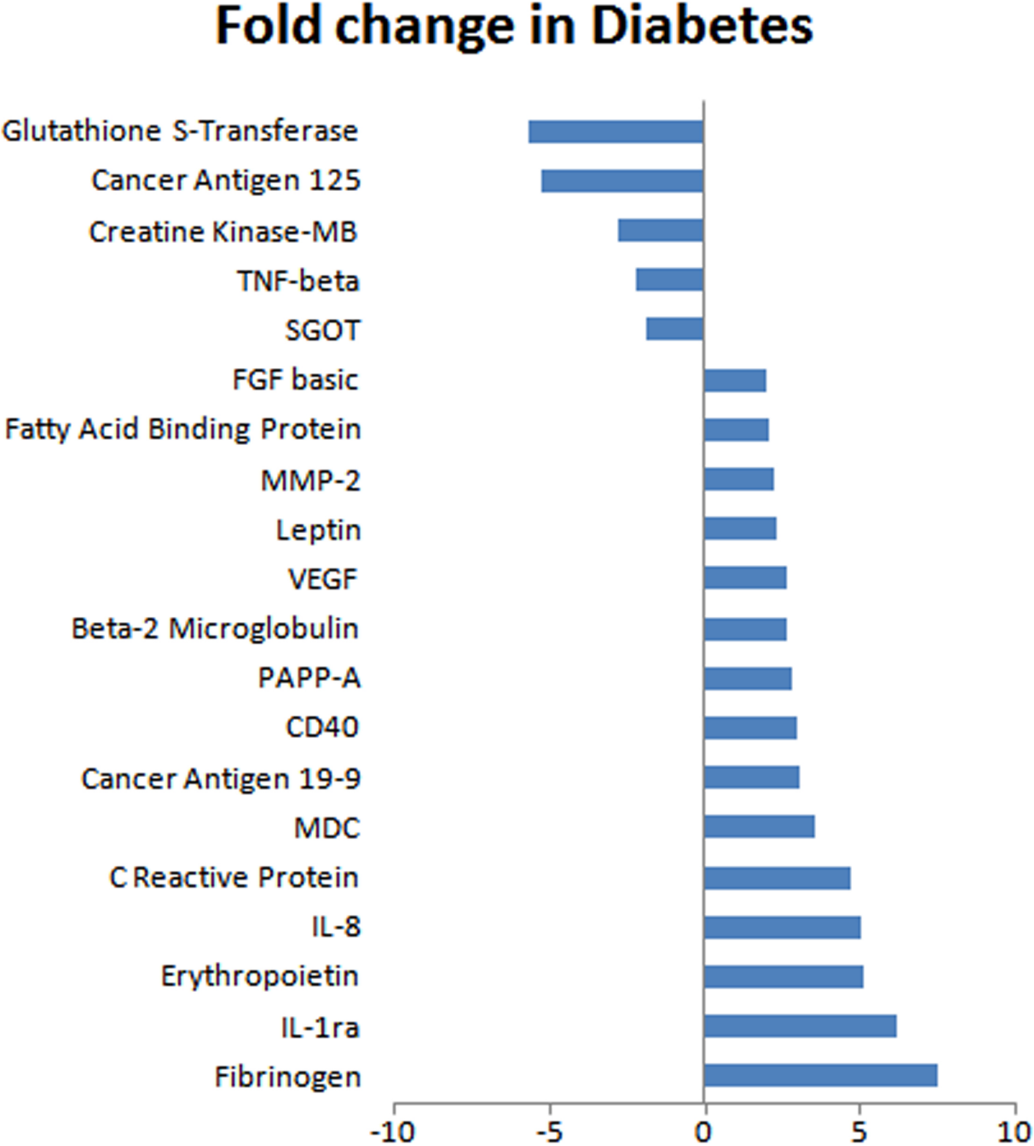
List of analytes that showed a twofold or more change in their levels in diabetic aqueous samples when compared to non-diabetic samples. The figure shows analytes that showed significant difference (2 fold or more; abscissa) between normal and the diabetic aqueous. The most prominent decrease was observed in glutathione S-transferase, and an increase in fibrinogen.

**Figure 2:**
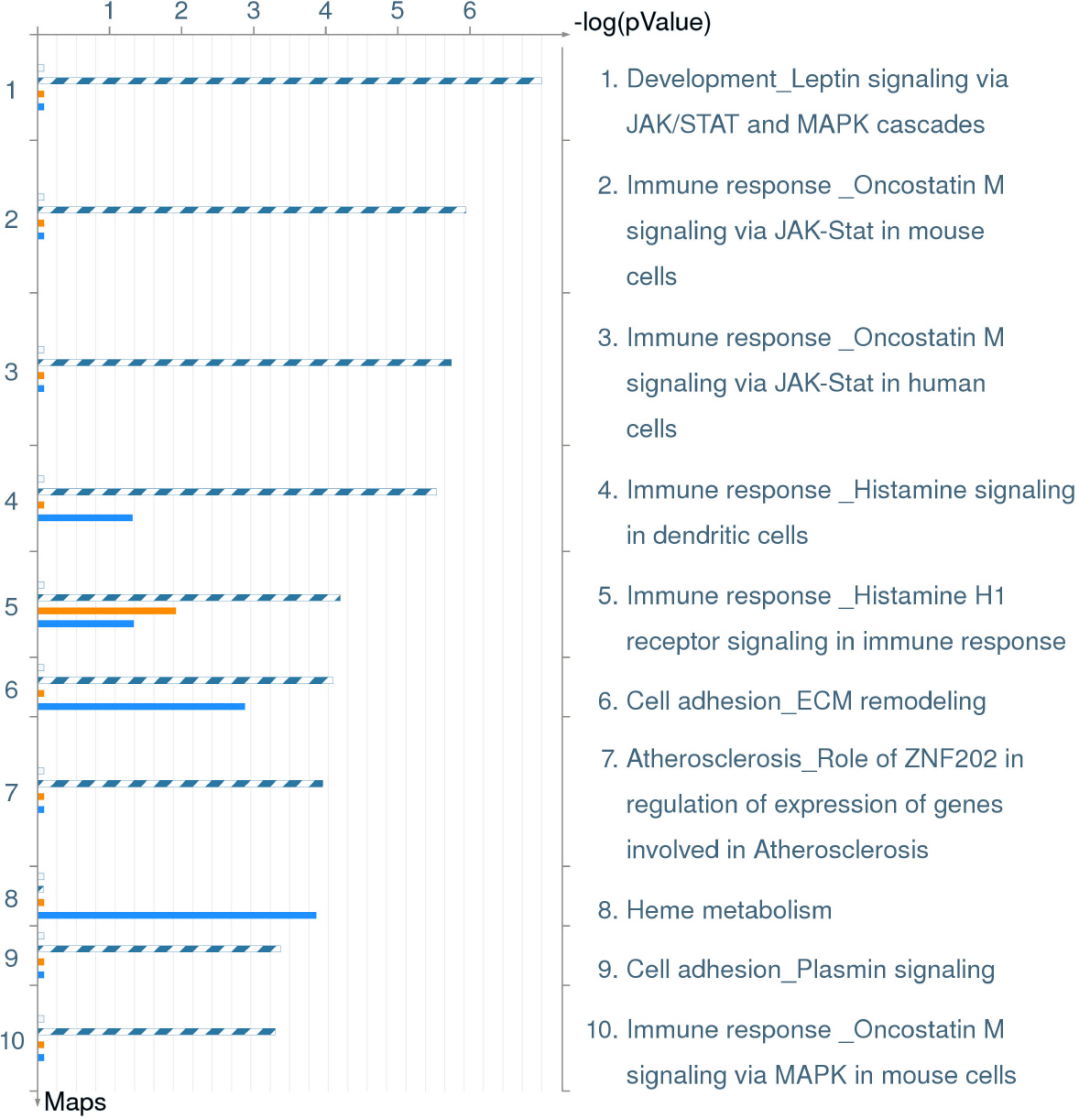
Distribution by canonical pathway maps in order of significance and their perturbation in diabetics. This figure shows canonical pathway maps, in order of significance and their perturbation in diabetics. Relevant to diabetes, the immune response and cell adhesion pathways were notably perturbed. Some pathways have been worked out in mouse cells and this is noted. The set of common analytes to both experiments is marked as blue/white stripes. The unique analytes are marked as colored bars (orange=diabetic, blue=normal/non-diabetic).

**Figure 3:**
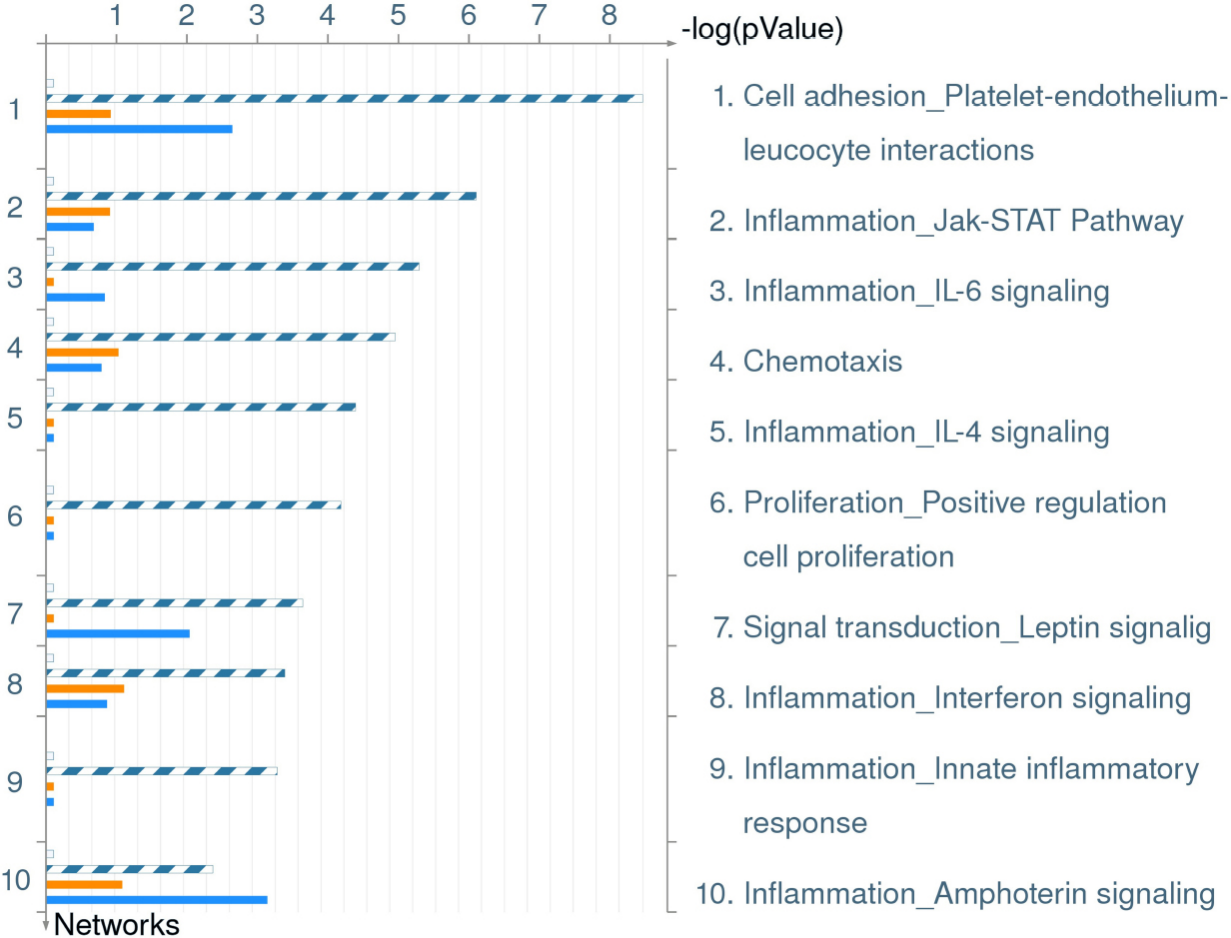
Distribution by GeneGo processes in order of significance and their perturbation in diabetics. This figure shows GeneGo processes in order of significance and their perturbation in diabetics. Relevant to diabetes, the cell adhesion, inflammation and chemotaxis pathways were notably perturbed. The set of common analytes to both experiments is marked as blue/white stripes. The unique analytes are marked as colored bars (orange=diabetic, blue=normal/non-diabetic).

**Figure 4:**
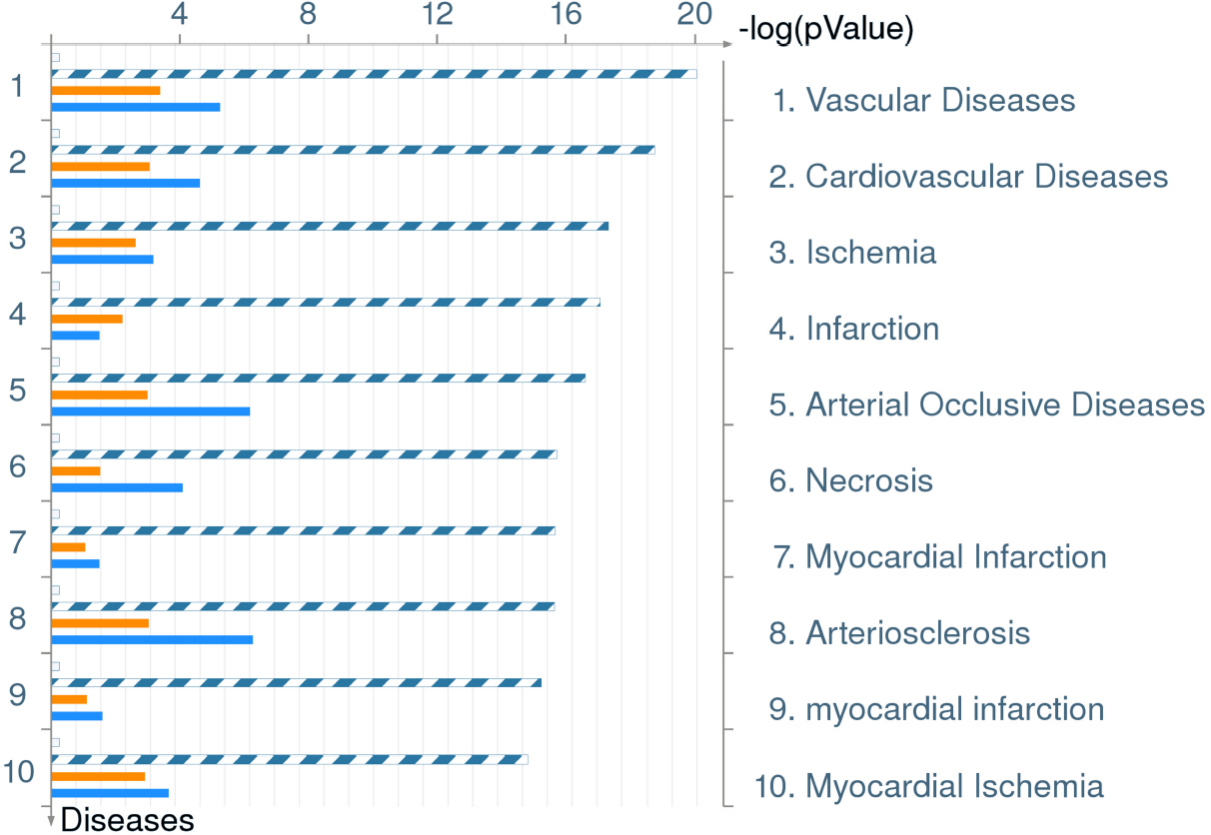
Distribution by disease biomarkers in order of significance and their perturbation in diabetics. This figure shows disease biomarkers in order of significance and their perturbation in diabetics. As expected in diabetes, biomarkers for vascular disease, ischemia and infarction showed perturbation. The set of common analytes to both experiments is marked as blue/white stripes. The unique analytes are marked as colored bars (orange=diabetic, blue=normal/non-diabetic).

The comparison identified significant differences between the two groups with several analytes showing upregulation and downregulation. Analytes that showed twofold or more changes are shown in Figure 1. There were analytes uniquely expressed in diabetic or normal samples. Comparison between processes in non-diabetic and diabetic aqueous are shown in Table 6. There were also changes in the canonical pathways (Figure 2). The top 10 most significantly altered GeneGo processes included the ones that involved cell adhesion (platelet-endothelium leukocyte interactions, inflammation, chemotaxis, proliferation [positive regulation], and signal transduction; Figure 3). The vascular diseases, infarction, arterial occlusion, ischemia, arteriosclerosis, and necrosis were the most significant diseases whose biomarkers were perturbed in diabetes (Figure 4).

**Table 6:** Comparison between processes in diabetic and non-diabetic aqueous humor.

**Number**	**Processes**	**Size**	**Target**	**Pathways**	**p value**	**zScore**
**Common**						
1	Locomotory behavior (42.6%), G-protein coupled receptor protein signaling pathway (51.1%), cell surface receptor linked signal transduction (66.0%)	50	13	10	8.58e−29	57.35
2	Response to external stimulus (56.2%), response to wounding (47.9%), response to stimulus (79.2%)	50	14	1	1.73e−31	61.78
3	Lipid transport (29.4%), sterol transport (23.5%), cholesterol transport (23.5%)	50	12	0	3.44e−27	57.78
4	Immune system process (54.0%), organ development (68.0%), response to stimulus (74.0%)	50	13	0	8.58e−29	57.35
5	Response to stimulus (75.0%), response to wounding (40.9%), response to external stimulus (47.7%)	50	7	6	4.60e−14	31.76
**Unique for Diabetes Samples**						
1	Response to external stimulus (50.0%), response to wounding (41.3%), cell surface receptor linked signal transduction (58.7%)	50	4	0	1.33e−11	51.55
2	Taxis (100.0%), chemotaxis (100.0%), locomotory behavior (100.0%)	5	1	0	6.01e−04	40.77
**Unique for Normal Samples**						
1	Response to wounding (37.2%), response to stimulus (69.8%), intracellular signaling cascade (51.2%)	50	8	0	1.68e−21	67.49
2	Response to external stimulus (53.8%), response to wounding (46.2%), cell surface receptor linked signal transduction (64.1%)	44	5	0	8.36e−13	44.93
3	Cellular di-, tri-valent inorganic cation homeostasis (100.0%), di-, tri-valent inorganic cation homeostasis (100.0%), cellular cation homeostasis (100.0%)	27	2	0	3.28e−06	37.72

The table lists the top related processes, the total number of genes in the network (Size), the number of genes in the network from the input list (Target) and the number of canonical pathways used to build the network (pathways). The comparison of diabetic and non-diabetic samples shows several processes listed under unique to diabetes and unique to normal samples changed in the diabetic and non-diabetic samples.

## Discussion

MAPs are a fully developed and validated technique [6,7]. Rule based medicine's (RBM’s) MAPs measure markers of cancer, infectious disease, autoimmunity, and cardiovascular risk (similar to age related macular degeneration [AMD] risk) as well as hormones, cytokines/chemokines, acute phase reactants, clotting proteins, growth factors, tissue modeling factors, and other typical plasma proteins [8]. A comprehensive analysis of an individual’s physiological status can be obtained by measuring a broad spectrum of biomarkers using MAPs. Luminex technology performs up to 100 multiplexed, microsphere-based assays in a single reaction vessel by combining optical classification schemes, biochemical assays, flow cytometry, and advanced digital signal processing hardware and software.

Multiplex analysis has been performed on aqueous samples in the past but with smaller numbers of analytes [9,10]. Our results demonstrate that with this technique around 100 different analytes can be successfully analyzed in small amounts of aqueous samples and provide base line values for 52 analytes. Many of the detected analytes such as CD40, C reactive protein, endothelin, basic fibroblast growth factor (bFGF), fibrinogen, glutathione S-transferase, matrix metalloproteinase (MMP)-3, tissue inhibitors of metalloproteinases (TIMP)-1, and vascular endothelial growth factor (VEGF) are implicated in several ocular pathologies, and therefore, their measurement could provide useful information [11-14]. Degradation of extracellular matrix proteins is regulated by MMPs. The activity of MMPs is in turn regulated by their natural inhibitors, TIMPs. MMPs and TIMPs play an important role in the pathogenesis of neovascularization in the retina. It is suggested that an imbalance between MMPs and TIMPs leads to neovascularization by affecting levels of VEGF, one of several genes associated with angiogenesis [15,16]. The role of VEGF in retinal as well as iris neovascularization is well established, and anti-VEGF therapy is becoming standard in the management of retinal neovascularization.

The MAP that we used was not specifically designed for ocular conditions. However, a specifically designed MAP for investigating ocular disease processes such as response of treatment (e.g., intravitreal injection of bevacizumab or steroids) can provide valuable and relevant information. It can also provide a method for subclassifying/staging diseases based on the pathophysiological processes.

We further analyzed the detected analytes to demonstrate the usefulness of global analysis in identifying cellular, biological, and disease processes to obtain an overall view of ocular physiology. Analytes represented a large number of pathways, albeit only a small number of proteins represented many of these. It should be noted that the MAP we used in this study did not represent all the cellular processes uniformly. Therefore, our data does not indicate if any cellular process is more prevalent in aqueous than another. We also did not make any attempt to compare the extent of any cellular process represented in the MAP with that expressed in the aqueous. The results demonstrate that aqueous analysis can provide useful information about cellular and disease processes. One of the limitations of using Metacore^TM^ or any other similar program is that the output is not specific for ocular diseases. Output of our results included disease processes such as myocardial infarction and cardiovascular diseases. It is because the program outputs any disease processes where the input analytes participate. Therefore, these results should be interpreted in the context of ocular physiology. At a molecular level, aqueous analysis could identify the canonical pathways and the associated gene networks. The MAP used in this study did not include analytes uniformly across all the cellular/disease processes. Therefore, the data cannot be taken as a representation of these processes in physiological/pathological conditions. Nevertheless, our results demonstrate that the functional processes can be studied using this technology. Moreover, application of specific MAPs can be created to suit specific needs.

Another potential use of such an analysis could be comparing two samples for a global view of changes in the functional processes associated with the ocular environment. Such information may be useful in understanding the pathobiology of a disease, assessing progression of a disease, or response to a treatment. The analysis of diabetic samples provided proof of principle for this hypothesis. The aqueous from diabetic patients showed differences in expected and relevant cellular as well as disease processes. Some processes relevant to diabetic retinopathy were found upregulated in diabetic aqueous samples including CD40, C reactive protein, erythropoietin, fatty acid binding protein, FGF basic, fibrinogen, IL-1ra, IL-8, leptin, macrophage-derived chemokine (MDC), MMP-2, and VEGF [17,18]. Equally relevant for diabetic pathophysiology were creatine kinase-MB, glutathione S-transferase, SGOT, and TNF-β, and they were downregulated in the diabetic aqueous samples [19,20]. It should be noted that since only two diabetic samples were used, changes observed in specific analytes may not be conclusive. Larger sample sizes need to be compared to confirm conclusions. However, this study suggests multiplex analysis may be a valuable global screening technique to identify pathways that warrant further investigation.
